# From Silos to Solidarity: Case Study of a Patient-Centered, Integrative Approach to Opioid Tapering and Chronic Pain Mitigation in a Multidisciplinary AIDS Clinic

**DOI:** 10.33696/AIDS.3.012

**Published:** 2021

**Authors:** S Pullen, VC Marconi, C del Rio, C Head, M Nimmo, J O’Neil, M Ziebart

**Affiliations:** 1 Department of Rehabilitation Medicine, Emory University School of Medicine, Atlanta, GA, United States; 2 Emory University School of Medicine, Department of Medicine, Division of Infectious Diseases, Atlanta, GA, United States; 3 Emory University Rollins School of Public Health, Department of Global Health, Atlanta, GA, United States

**Keywords:** HIV, AIDS, Chronic pain, Opioids, Physical therapy

## Abstract

**Background:**

People with HIV (PWH) are at a disproportionate risk for experiencing both chronic pain and opioid use disorder (OUD). Prescription opioid tapering is typically addressed within the “silo model” of medical care, whereby attention is focused solely on opioid addiction rather than also addressing chronic pain management, and limited communication occurs between patient and providers.

**Objective:**

This descriptive case study examined an integrative, collaborative care model consisting of Provider, Physical Therapist (PT), and Patient aimed at decreasing chronic pain and opioid use within a multidisciplinary HIV/AIDS clinic.

**Method:**

A physical-therapy based model of chronic pain mitigation and physician-driven opioid tapering was implemented. The Provider, PT, and Patient worked collaboratively to address physiological pain, pain coping skills and opioid tapering. A patient case example was used to illustrate the implementation of the model for a future, larger study in the same patient population.

**Results:**

This model was feasible in this case example in terms of clinic workflow and acceptability to both the Patient and Providers in this clinic. After the intervention, the Patient’s pain was fully eliminated, and he had ceased all opioid use.

**Conclusion:**

Results of this case study suggest that utilizing an integrative, patient-centered approach to both chronic pain management and opioid tapering may be feasible within the context of a multidisciplinary HIV/AIDS clinic. Generalizability is limited by case study model; however, this gives insight into the value of a collaborative alternative compared to a “silo” model of opioid tapering and chronic pain management in preparation for a larger study.

## Introduction

Chronic pain, defined as pain lasting longer than three months, is a significant public health burden and has been associated with depression, anxiety, poor quality of life and opioid use disorder (OUD) [1]. The standard of care for chronic pain management in the United States has historically emphasized pharmacological management, leading to elevated usage of opioid analgesics over the last several decades and increased prevalence of OUD and overdose deaths [2–4]. Despite this, there has been a concurrent 25% increase in reports of adults with chronic pain over the last two decades, indicating that increased opioid use does not correlate with decreased pain [5]. In fact, the use of opioids for chronic pain-while pervasive remains controversial, as there are not data to support the effectiveness of opioids in the mitigation of non-malignant chronic pain [4,5].

Compared to the estimated 8-20% of the general US population that reported chronic pain, the prevalence of chronic pain in people with HIV (PWH) is estimated to be as high as 85% [6–9]. The etiology of chronic pain for PWH is multifactorial resulting from HIV-related causes (virally-mediated nerve damage, chronic inflammation, side effects from antiretroviral therapy (ART)), psychological exacerbators (depression, anxiety, post-traumatic stress disorder), and other factors such as socioeconomic stressors or musculoskeletal injuries [10–14]. Among PWH, chronic pain has been associated with psychological and functional morbidity, decreased use of ART, and reduced retention in HIV primary care [15,16]. Prescription opioid use is also higher among PWH than the general population and is also associated with suboptimal adherence to ART [17–19].

In accordance with the HIV Medical Association’s (HIVMA) clinical practice guidelines for the management of chronic pain, opioid analgesics should not be the first line of defense against chronic pain; rather, non-opioid drugs, as well as non-pharmacological and biopsychosocial treatments, should be the initial approach [16,20]. A recent body of research supports physical therapy (PT) interventions such as therapeutic exercise, manual therapy, and patient education as an effective, cost-efficient, low-risk treatment for acute and chronic pain. Furthermore, preliminary studies suggest that when chronic pain is managed through skilled PT, PWH report a decrease in pain levels and use of analgesics as well as an increase in functional independence and quality of life [7,21–26]. In order to decrease opioid use and safely initiate opioid tapering, recent recommendations from the Centers for Disease Control and Prevention (CDC) have called for an integrative approach to pain management and opioid tapering [1]. This involves coordination of the care team between the primary care clinician, mental health provider, and other specialists, including PT, to optimize nonopioid pain management [1]. However, a “silo” model is still utilized within most healthcare systems, often leading to fragmented care with sub-optimal communication between providers and patients [27–29].

Feasibility studies are broadly defined as preliminary studies done before a larger trial that ask “*whether something can be done, should we proceed with it, and if so, how*” [30]. This case study was designed to determine the feasibility of a larger study using an integrated approach to opioid tapering as a framework for chronic pain mitigation and OUD prevention at a multidisciplinary HIV clinic in Atlanta, Georgia, USA.

## Methods

The study took place at the Ponce de Leon Center, one of the largest and most comprehensive ambulatory outpatient HIV/AIDS facilities in the nation, attending to underserved and at-risk populations in metro Atlanta, Georgia. Out of the 6200 enrolled HIV-positive patients, approximately 90% identify with under-represented minority groups, 70% live below the federal poverty level, 42% are uninsured, and 26% receive Medicaid. Of metropolitan cities in the United States, Atlanta ranks second for incidence and prevalence of HIV, and more than 70% of PWH in Atlanta reside within two miles of the Ponce Center, an area recognized as a spatial clustering of the Atlanta HIV epidemic [31,32]. Our integrative pain management model, consisting of a primary HIV physician, PT, and the Patient, exemplifies the type of collaboration required to address the complex, multi-faceted issues of chronic pain and opioid use among PWH.

In this descriptive case study, we illustrate the experience of a Patient and his clinical team (PT and Provider) within this integrated model. The inclusion criteria for this case study was an HIV-positive adult with a chronic pain diagnosis and who was currently taking prescribed opioid analgesics. The Patient gave consent to the intervention. The Patient was a 50-year-old male who was diagnosed with HIV in 2002. At the time of this study, the Patient’s ART regimen consisted of emtricitabine-tenofovir AF (Descovy), and darunavir ethanolate (Prestiza)/ritonavir (Norvir). His CD4 count remained above 500 cells/mm^3^ and his viral load remained undetectable throughout the intervention. The Patient had a past medical history significant for avascular necrosis (AVN) of the right femoral head followed by right total hip arthroplasty with subsequent post-operative septicemia (2017), AVN of the right shoulder (2014), anal cancer (2016), Disseminated Mycobacterium Avium (DMAC) Complex, Cytomegalovirus (CMV) pneumonitis, controlled diabetes mellitus, and adrenal insufficiency. While there were no documented mental health diagnoses in the Patient’s chart, he did express some anxiety surrounding his chronic pain and the opioid tapering. These concerns were addressed by both the MD and the PT by providing support and encouragement, however the Patient declined suggestions for mental health counseling at the clinic. The Patient had stable food and housing, living with his husband in a single-family home. He was initially referred for a 30-year history of right scapular and shoulder pain secondary to a traumatic blunt-force injury in which a piece of wood fell off a building and onto his back. The Patient had been on varying prescriptions and opioid analgesics for nine years preceding this study, detailed in Table 1 by drug name, years taken, and subsequent morphine milligram equivalents (MME), which equate different opioid doses into one standardized value to allow for risk assessment and comparison between various opioid medications [33]. At the time of evaluation, the Patient reported taking 100mg of tramadol six times a day (MME=60) for the preceding five months to manage chronic pain.

Prior to study initiation, the Patient, Provider, and PT mutually agreed upon the planned intervention of PT, opioid tapering, and pain management. At the initial physical therapy visit, the Patient and PT discussed the Patient’s plan of care, consisting of targeted, pain-site based manual therapy/massage, therapeutic exercise, pain coping strategies, and Transcutaneous Electrical Nerve Stimulation (TENS). TENS is an evidence-based, non-invasive, non-pharmacological treatment for acute and chronic pain, delivered to the Patient through surface electrodes placed on the pain site [34]. The resulting deep tissue stimulation relieves pain by both peripheral and central mechanisms, with analgesia activated at opioid and α-2 noradrenergic receptors (peripherally) and at sites in the brainstem and spinal cord that utilize opioid, serotonin, and muscarinic receptors (centrally) [34]. The Patient’s individualized therapeutic exercise/home exercise program, manual therapy, and TENS application schedule can be found in Table 2. The Patient’s tramadol tapering followed CDC guidelines and was under direct Provider supervision and approval in conjunction with the Patient and PT.

Pre- and post-intervention data were collected via the following three instruments and their corresponding scoring criteria: 1) the Brief Pain Inventory (BPI) [35], a self-reported measurement tool used to clinically evaluate pain, including severity, interference with activities of daily life, and percent relief afforded by pain treatments and/or medications; 2) the 36-Item Short Form Survey (SF-36) [36], a widely validated tool that measures quality of life through eight health concepts: physical functioning, bodily pain, role limitations due to physical health problems, role limitations due to personal or emotional problems, emotional well-being, social functioning, energy/fatigue, and general health perceptions; and 3) the 0-10 numeric pain rating scale (NPRS) [37]. The Patient was seen primarily for in-person clinic visits (n=6) and one telehealth appointment via phone call. This case study was part of an Institutional Review Board -approved protocol from the author’s home institution and both oral and written informed consent were obtained from the Patient prior to study initiation. Data was de-identified, recorded and stored in a secure digital, password protected database.

Pain coping skills were taught to the Patient and followed three self-management techniques upon the onset of pain, before taking an opioid analgesic: 1) Perform individualized home PT stretching/exercise routine; 2) Place the TENS unit electrodes on the painful area for 20-30 minutes; 3) Perform diaphragmatic/paced breathing with the following instructions: “*Take a deep breath in slowly through your nose, letting your chest and lower belly expand. Breathe out slowly through your mouth with slightly pursed lips. Focus on the sound and the feeling of your chest, belly, and body filling with air, then letting go.*” Each visit began with the Patient and PT reviewing the prior week’s pain, fidelity to home exercise program, and opioid usage. At each visit, the PT performed manual therapy in accordance with the Patient’s pain sites and progressed the home exercise program, ending with a review of pain coping skills. The Patient and PT were present in-person for all 7 visits, the Provider, PT and Patient were present for 2 in-person visits, and the Provider was apprised via email or telephone of the Patient’s status weekly. Opioid tapering was advanced only once the Patient, Provider, and PT discussed - as an equally weighted team - the best way to progress given the Patient’s readiness, PT-driven pain mitigation plan, and Provider-driven safe tapering guidelines.

## Results

Pre- and post-intervention outcomes were measured through the Numeric Pain Rating Scale (NPRS), Brief Pain Inventory (BPI), SF-36 scores, and opioid use/MME. Subjective information was gathered verbally at each session, and opioid prescriptions were confirmed through the Patient’s provider and electronic medical charts. The Patient had ceased all opioid use at the end of visit eight. However, the Patient, PT, and Provider met three additional times to establish a proper medication regime for persistent neuropathic pain secondary to peripheral neuropathy. Post-intervention, the Patient had a 100% reduction in pain (from 4/10 to 0/10) showing a minimal clinically important difference (MCID) for chronic pain, which is defined as a decrease of 1.7 points or 27.9% decrease on the NPRS [38]. While his pain reports did decrease throughout the intervention, he also experienced noteworthy fluctuations that speak to the complexity of chronic pain. His decrease in MME was well above CDC’s guidelines for “safe and successful” tapering, defined as a 25% decrease in MME [1]. Table 3 details the Patient’s opioid tapering schedule, pain reports, and PT goal realization during the intervention.

SF-36 scores were recorded at the Patient’s initial clinical evaluation and after the intervention (Table 4). Scores in all domains improved by the final visit, and the Patient’s pre-intervention (M=46.33, SD= 14.59) and post-intervention (M=75.28, SD=32.41) SF-36 scores showed statistical significance in all categories (p=0.0186, CI=95%).

Additionally, changes in the Patient’s BPI scores occurred in each domain between pre- and post-intervention, which most notably identified a decrease in pain severity, decreased pain interference, and increased pain relief.

## Discussion

Feasibility studies are broadly defined as preliminary studies done before a larger study that ask *whether something can be done, should we proceed with it, and if so, how?* [30]. This case study was completed to assess the feasibility of a larger study examining pain mitigation and cessation of opioid use in a multidisciplinary HIV/AIDS clinic. In this Patient example, this model was acceptable to Patient, clinical providers and clinic workflow. This case was unique in the close communication and teamwork of the Patient, Physical Therapist, and Provider with the shared goal of pain reduction and opioid tapering. The Provider’s expertise in medical management of HIV and prescription analgesics was essential to safely tapering the Patient’s opioids in the context of HIV-related comorbidities and current ART regimen. Equally, the physical therapist’s expertise in physiological chronic pain management was pivotal to creating a personalized pain mitigation program. The patient-centered intervention with the Patient as an equal member of the care team was fundamental to both goal attainment and Patient empowerment. Finally, the courage, resilience and dedication demonstrated by the Patient throughout the intervention was a key part of the successful outcome.

The success of the interdisciplinary approach was especially evident at the conclusion of the study. While the Patient’s chronic, musculoskeletal neck and shoulder pain subsided and eventually disappeared, the Patient’s distal, symmetrical polyneuropathy, a painful and common long-term side effect of both HIV and ART, remained. While the Patient had effectively tapered off of opioids, his ongoing neuropathic pain required a shift to neuropathic-based, non-opiate analgesia. Two neuropathic medications were subsequently trialed after the Patient completed the study. Pregabalin (Lyrica) was found to be most effective for the Patient’s neuropathy, and the Patient remains on this at the time of publication.

The feasibility of this case study was attainable partially due to the clinical communication at a multidisciplinary HIV/AIDS clinic, with all clinical services on-site and with all providers in close contact. If a patient is receiving care at several different locations (or silos), this integrated approach could be limited as interdisciplinary care is not traditionally a part of the core framework. Despite this, many aspects of the interdisciplinary approach can be used even when a patient receives their care at distinct clinical sites. Increased provider communication – regardless of the physical distance between providers and their patients – will vastly improve both clinical outcomes and trusting relationships.

Currently, one of the barriers to successful opioid tapering is that the sole focus is the substance use with little or no focus on the treatment of unremitting pain for which the Patient was originally prescribed opioids. Medication-assisted treatment (MAT) - the use of medications for the treatment of opioid disuse - has proven to be clinically effective and reduce the need for inpatient detoxification programs [39]. While this is promising from an addiction and overdose standpoint, MAT program completion may still leave individuals suffering from chronic pain without any relief alternative once they are no longer taking opioids. In order to fully address the complexity of a patient’s chronic pain and subsequent opioid use, we must approach both the pain and opioid use from a multidisciplinary approach that equally considers addiction, physiological pain and psycho-social comorbidities.

**Figure 1:**
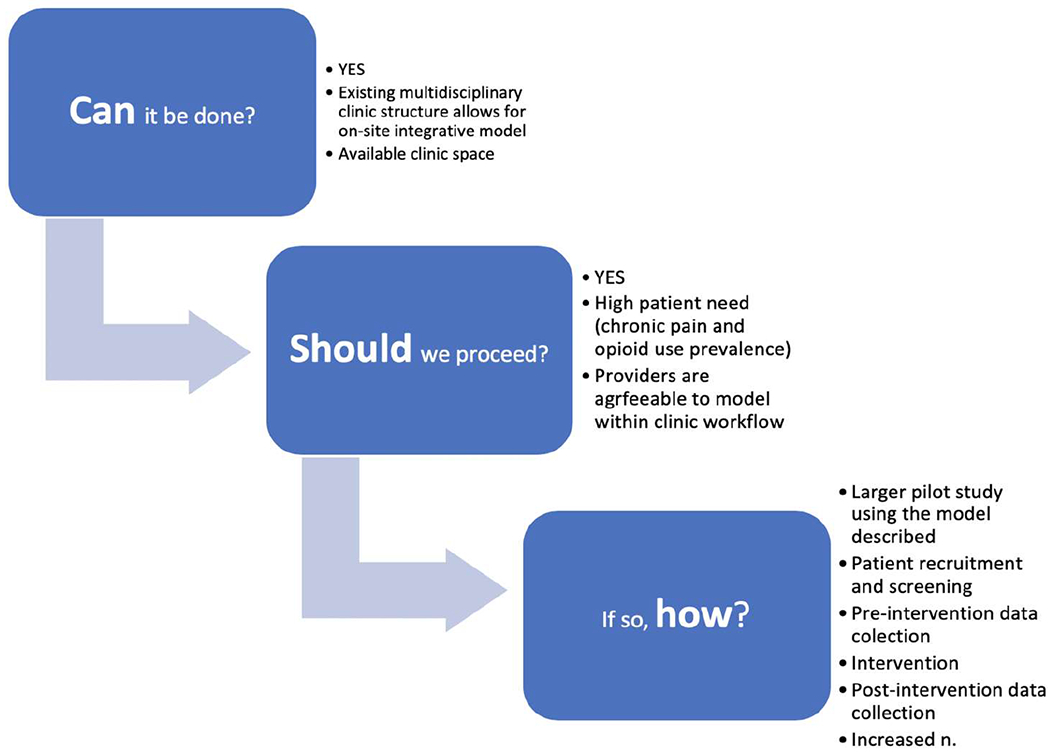
Feasibility of an integrated approach to opioid tapering and pain management in a multidisciplinary HIV clinic.

This case study demonstrated that an integrated approach to chronic pain management and opioid weaning could be a feasible model within the context of a multidisciplinary HIV clinic setting (Figure 1). The target outcome from this study was not solely to reduce opioid use but to manage chronic pain effectively so that the opioids were no longer necessary. This model advances the type of collaboration required to address the complex, multi-layered issues of chronic pain and opioid use, and needs to be trialed in a larger study. By advancing an alternative to the overused, perilous, and ultimately ineffective prescription opioid analgesics, we aim to cultivate the type of patient-centered collaboration that is our best hope for preventing the devastating consequences of OUD and overdoses. To fully address the complexities of chronic pain and opioid use mitigation among PWH, we must aim to bridge the existing silos which impede holistic recovery in this unique patient population.

## Figures and Tables

**Table 1:** Past Opioid Prescriptions and MME.

Drug Name	Years prescribed	MME Range
MS Contin	2013-2019	90/60/120/80
Percocet	2012-2017	30
Tramadol	2011-2019	15-60

**Table 2:** Participant therapeutic exercise, home exercise program, manual therapy, TENS application.

Therapeutic exercise/home exercise program	Stretching to subscapular region, upper trapezius, sternocleidomastoid, Bilateral shoulder external rotation strengthening
Manual therapy	Soft tissue massage to bilateral upper trapezius, levator scapulae, rotator cuff
TENS	The Patient was introduced to TENS at the initial visit and received a home unit on the second visit. Patient instructed on proper placement of pads and intensity level directed by the Patient’s comfort.

**Table 3:** Opioid Tapering, Pain Scores and Goals Met.

Treatment Session #	Opioid Prescription (Pills/Month)	Daily Opioid Use (Tramadol 100 mg) (MME)	Average Pain Score	Opioid Schedule	Goal Met
Initial Evaluation	90	6 Tramadol per day (60 MME)	4/10	Breakfast, lunch, dinner	
1	90	6x per day (60 MME)	2/10	Breakfast, lunch, dinner	
2	90	6x per day (60 MME)	2/10	Breakfast, lunch, dinner	
3	90	3x per day (30 MME)	4/10	Breakfast, lunch, dinner	
4	90	3x per day (30 MME)	3/10	Breakfast, lunch, dinner	
5	90	2x per day (20 MME)	0/10	PRN, attempting to only take in the PM	1. Neck/shoulder pain is decreased with TENs unit or self-massage
6	90	1x per day (10 MME)	0/10	In the PM, PRN	
7	90	(0 MME)	0/10		2. Shoulder/neck pain is resolved
8	0	0 MME	0/10		3. Independent with home exercise program

**Table 4:** Change in SF-36 Score by Domain.

Measure	Baseline	Final
Physical Functioning	65	75
Role limitations due to physical health	25	0
Role limitations due to emotional problems	33	100
Energy/fatigue	30	60
Emotional well-being	44	100
Social Functioning	50	100
Pain	55	67.5
General health	65	75
Health change	50	100
